# Ethnomedicine use in the war affected region of northwest Pakistan

**DOI:** 10.1186/1746-4269-10-16

**Published:** 2014-01-31

**Authors:** Muhammad Adnan, Ihsan Ullah, Akash Tariq, Waheed Murad, Azizullah Azizullah, Abdul Latif Khan, Nawab Ali

**Affiliations:** 1 Department of Botany, Kohat University of Science and Technology, 26000 Kohat, Pakistan; 2 Department of Biological Sciences and Chemistry, University of Nizwa, Nizwa, Oman; 3 Department of Biotechnology and Genetic Engineering, Kohat University of Science and Technology, 26000 Kohat, Pakistan

**Keywords:** Indigenous knowledge, Traditional medicines, Medicinal plants, Diseases

## Abstract

**Background:**

North-West of Pakistan is bestowed with medicinal plant resources due to diverse geographical and habitat conditions. The traditional use of plants for curing various diseases forms an important part of the region’s cultural heritage. The study was carried out to document medicinal plants used in Frontier Region (FR) Bannu, an area affected by the “War on Terror”.

**Methods:**

Fieldwork was carried out in four different seasons (spring, autumn, summer and winter) from March 2012 to February 2013. Data on medicinal plants was collected using structured and semi-structured questionnaires from 250 respondents. The voucher specimens were collected, processed and identified following standard methods.

**Results:**

Of the 107 species of ethnomedicinal plants reported, fifty percent species are herbaceous. The majority of the reported species were wild (55%) but a substantial proportion are cultivated (29%). For most of the plant species (34%), leaves are the most commonly used part in the preparation of ethnomedicines. The most common use of species is for carminative purposes (14 species), with the next most common use being for blood purification (11 species). The main methods used in the preparation of ethnomedicinal recipes involves grinding and boiling, and nearly all the remedies are taken orally along with ingredients such as water, milk or honey for ease of ingestion. Traditional healers prepare plant remedies using one or more plants. There was a significant correlation (r^2^ = 0.95) between the age of local people and the number of plants known to them, which indicates that in the coming 20 years, an approximate decrease of 75% in the indigenous knowledge may be expected.

**Conclusion:**

Traditional medicines are important to the livelihoods of rural communities in the region affected by the Global war on Terrorism. The medicinal recipes are indigenous; however, there is a threat to their future use on account of rapid modernization and terrorist activities. Documentation of medicinal plants and recipes may help in the conservation of the regional indigenous medicinal knowledge for future generations and to provide a baseline for further studies.

## Introduction

Plants have been used as folk medicine all over the world for centuries [1] and indigenous communities have developed their own specific knowledge on plant resources, uses, management and conservation [2]. Ethnomedicinal treatment is not merely a medical system but part of a culture [3]. Today, around 25% of all prescribed medicines in the developed world contain ingredients derived from medicinal plants [4]. It has been estimated that herbal medicines are used by more than 80% of the world’s population in developing countries to meet their primary healthcare needs [5]. The traditional use of plants and plant resources is rapidly increasing due to their minimal side-effects and (affordable) accessibility, and because they sometimes represent the only source of healthcare available to poorer communities [6]. However, the key issue in the current era is the loss of indigenous medicinal plant and preparation knowledge, which can serve as a guideline on plant-based therapeutic research for many scientists around the world.

Pakistan has a diverse flora containing a total of 1,572 genera and 5,521 species, most of which are confined to the Hindukush, Himalaya and Karakorum regions [7,8]. People collect about 600 medicinal plant species as one of the major non-timber forest products (NTFPs) [9]. Of these species, 500 are commonly used in traditional healthcare practices and 350 are traded for millions of US dollars to national and international markets [10]. Twenty-eight leading herbal manufacturing units use medicinal plants for making various preparations, which include 75 crude herbal drugs that are extensively exported. About 60,000 traditional practitioners (Hakeems) in rural and remote areas utilize medicinal plants as household remedies for curing several diseases [11]. Local communities have centuries of traditional knowledge and practice relating to plants of their regions that have been transmitted from generation to generation [12]. About 84% of the country’s population was dependent on traditional medicines in the early 1950’s [13]; however, the practice is now confined to remote areas [14].

This study has been carried out in the Frontier Region (FR) Bannu, which has suffered heavily due following the onset of the Global War on Terrorism. Various ethnomedicinal studies have been carried out [12,15-18] in other regions of Pakistan; however, the FR has yet to be explored due to limited access. The area represents one of the country’s richest centers of biodiversity and it is a strong source of indigenous knowledge. Most of the population of the area is rural with a low literacy rate; hence they are more dependent upon natural resources, and especially on plants for their healthcare needs and livelihoods. War has crippled modern health facilities in the study area, which in turn has resulted in the spreading of gastrointestinal and skin related diseases among others. However, local people are increasingly using ethnomedicines to treat such diseases at the local level. Shinwari *et al*. [12] perceived a diminishing of indigenous knowledge due to the ever increasing influence of global commercialization and socio-economic transformation, and a dire need was expressed to preserve such knowledge on medicinal plants before it disappears. Hence, the present study was designed with the following objectives: (i) to identify and explore plant species that are being used locally for the treatment and prevention of various diseases; (ii) to document traditional recipes from medicinal plants including methods of preparation and modes of administration; and (iii) to investigate the current and future status of traditional knowledge among different age groups. The present study may help in the preservation of indigenous knowledge on ethnomedicines and provide baseline data for future studies.

## Materials and methods

### Study area

**Figure 1:**
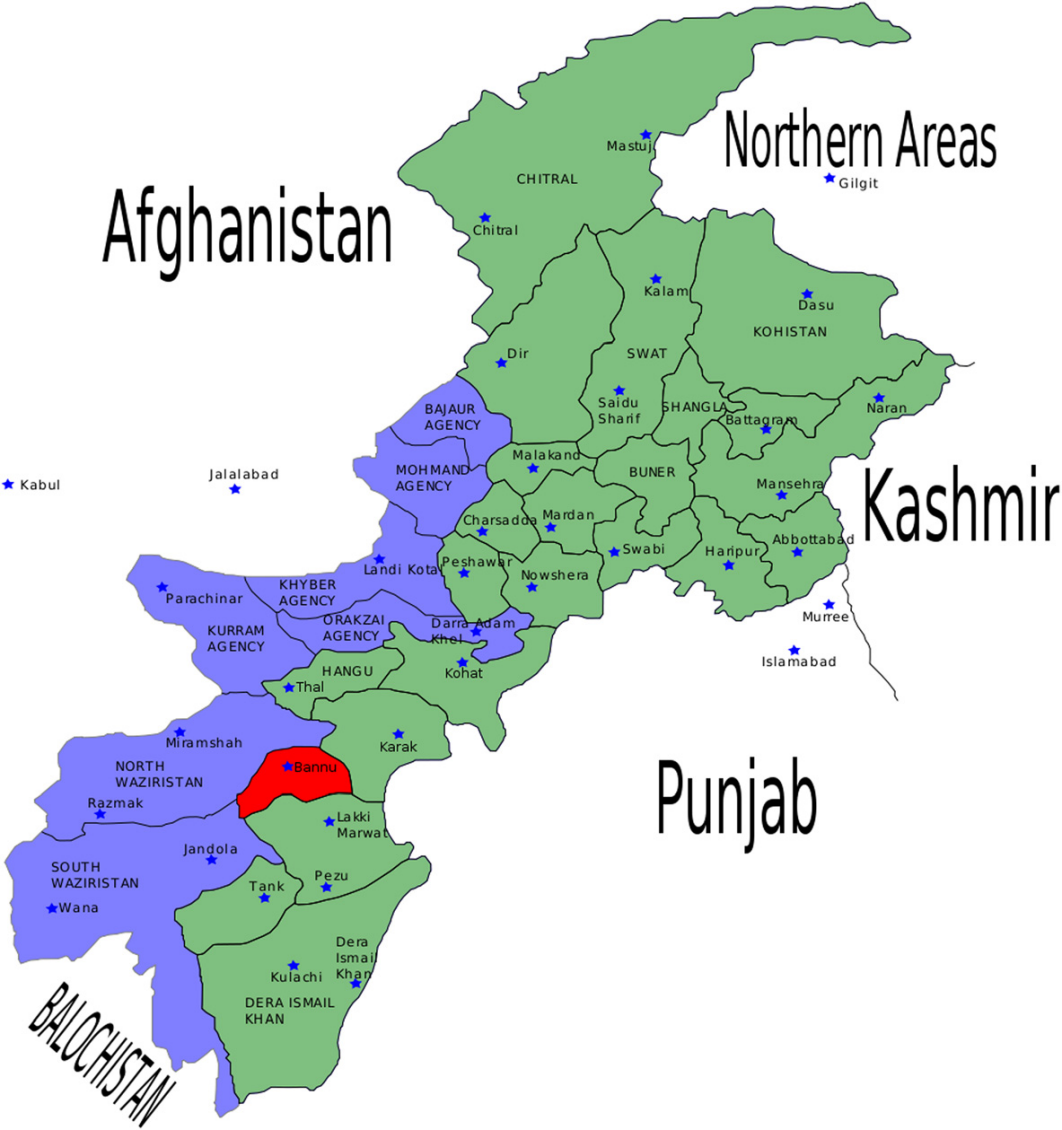
Map of the study area.

The present study was carried out in the Frontier Region (FR) Bannu located in the south of Khyber Pakhtunkhwa (KPK) Province, Pakistan (Figure 1). FR-Bannu consists of a total area of 877 square kilometers with a population of 19,593. The area lies within the Karakoram mountain range [19] between 32°-43 to 33°-06 N latitude and 73°-20 to 70°-07 E longitude. The total cultivated area is about 33,000 acres, with wheat, maize and sugarcane being the main cultivated crops [20]. About 25% of the inhabitants of the area as well as Afghan refugees are engaged in the collection and marketing of medicinal plants [20]. The area consists of alluvial plain with an annual rainfall of 111.36 mm [21]. The dominant plant species are *Acacia modesta, Acacia nilotica, Calotropis procera, Dodonaea viscosa* and *Withania somnifera*.

### Sampling

Ethnomedicinal data was collected in four field visits per season in spring, autumn, summer and winter from March 2012 to February 2013. The methods for the collection of data and voucher specimens during the field study followed that described by Martin [22]. Initially, local administrative officers and representatives (Malik) of the study area were visited, who provided information on key resource persons in the field of medicinal plants. The key persons then suggested 269 potential respondents (local healers, rural herbalists and elders), all of whom were known in the community for their knowledge on medicinal plants and ethnomedicines. Prior to data collection, a brief group discussion was held with the respondents in order to gain their consent, to explain objectives of the research study and to assure them protection of their traditional knowledge. This was done to clarify the purpose of the research and to build confidence among respondents so that they would provide reliable information without holding any suspicion. Among the respondents, 19 were reluctant to provide any information leaving a total of 250 respondents being selected for data collection.

The biographic characteristics of the respondents in this study include age, gender and ethnic group. Of the 250 respondents, 201 were male and 49 female, and all belonged to one of three ethnic groups (*Wazir*, *Banuchi* or Afghan refugees). Due to customary restrictions in the study area, it was difficult to identify and gain access to a large number of females with knowledge on medicinal plants. The majority of the respondents were aged between 35 and 90 years and most (133) were *Waziri*, the largest ethnic group of the study area (Table 1). All the respondents and focal persons of the study area provided permission to publish and protect the data on traditional medicines provided by them.

**Table 1:** Age groups, number of interviews and male to female ratio of various ethnicities

**Age groups**	**No. of interviews**	**Wazir**	**Banuchi**	**Afghan refugees**
		**(Male, female)**	**(Male, female)**	**(Male, female)**
11-20	7	4 (4, 0)	2 (2, 0)	1 (1,0)
21-30	10	5 (5, 0)	3 (3, 0)	2 (2,0)
31-40	18	9 (7, 2)	6 (5, 1)	3 (2, 1)
41-50	30	17 (14, 3)	8 (7, 1)	5 (3, 2)
51-60	52	30 (26, 4)	15 (12, 3)	7 (5, 2)
61-70	60	36 (31, 5)	16 (10, 6)	8 (6, 2)
71-80	43	22 (19, 3)	17 (12, 5)	4 (4, 0)
81-90	30	10 (7, 3)	12 (9, 3)	8 (5, 3)
Total	250	133 (113, 20)	79 (60,19)	38 (28,10)

### Identification of medicinal plants used as ethnomedicines

Structured and semi-structured questionnaires were designed for data collection. For this purpose, individual interviews were held with each respondent in the local language of *Pashto*, which is spoken by all ethnic groups in the study area. More structured questionnaires were used to obtain specific information about medicinal plants of the study area, and informants were asked about the number of plants known to them, their local names, life-form, medicinal uses, occurrence, plant status and the most preferred part used for treatment [23]. Plant life-form implied herb, shrub or tree. Plant use referred to the use of either part of the plant or several parts and were categorized accordingly as whole plant, leaves, roots, bark, flowers, seeds, shoots, rhizomes and fruits. Plant status referred to the classification of plants across three categories, i.e. wild or cultivated or both.

Specimens of recorded medicinal plants were collected in their flowering and fruiting seasons during field visits and were processed in the laboratory using normal methods [22,24]. Specimens were identified with the help of a plant taxonomist in the Department of Botany, Kohat University of Science and Technology (KUST). Following plant identification, specimens were pressed and dried in blotting sheets. Before mounting on the herbarium sheets, the plants were treated either with formaldehyde or mercuric chloride solution (0.5%) in order to protect plant material from insect or fungal damage. The scientific names, family names and names of publication authors were corrected according to the flora of Pakistan and software index kewensis [8,25]. Identified plant specimens on herbarium sheets were placed in the herbarium at KUST.

### Documentation of ethnomedicinal recipes

Semi-structured questionnaires were used to gather information on the mode of preparation and administration as well as information on typical compliments used with the ethnomedicines. The ailments treated were grouped into 21 types including febrifuge, carminative, stomach problems and chest infections among others. Questions on ethnomedicines inquired on modes of preparation (powder, boiled etc.), administration (oral or dermal), use of single or mixture of plants, ease of intake and other ingredients used such as water, sugar, milk and others. All the data collected through structured and semi-structured questionnaires were organized using Microsoft Excel.

### Traditional knowledge and associated threats

Correlation between the age of the respondents and the number of medicinal plants known to them was analyzed using Pearson Correlation in SPSS [26]. Moreover, a conceptual diagram was developed in which the age groups in possession of indigenous knowledge were categorized and displayed showing current numbers (2013) and predicted numbers for 2023 and 2033. Data on the current status of indigenous knowledge was gathered during the present study while data for the next 10 and 20 years were predicted on the basis of a study by Shinwari [12], who predicted a 50% decline in indigenous medicinal plant knowledge among rural people for every 10 years due to modernization.

## Results and discussion

The present study has recorded valuable ethnomedicinal knowledge from an area almost inaccessible due to the current armed conflict. Indigenous people from different localities have their own specific knowledge on the traditional utilization of medicinal plants [2] and our study highlights certain threats related to the disappearance of such knowledge. Medicinal plants represent a significant contribution to human health and it has been suggested that their use is one of the most significant ways in which humans directly reap the benefits provided from biodiversity [27].

### Medicinal plants of the study area

In the war affected region of Pakistan, the use of folk remedies is a common practice and locals are highly dependent on the native flora for their healthcare needs. The inhabitants of the region use 107 plant species distributed across 90 genera and 56 families for the treatment of various ailments (Table 2). Such widespread use of medicinal plants for primary healthcare needs in the study area might be due to the lack of health facilities as a result of both increased armed conflict as well as cultural beliefs of the people who use ethnomedicines in the rural areas.

**Table 2:** Medicinal plants and uses

**Serial no.**	**Botanical names**	**Family**	**Local names**	**Life form**	**Part used**	**Plant occurrence status**	**Medicinal uses**	**Recipes**
1	*Acacia arabica* (Lam.) Willd.	Mimosaceae	Kekar	Tree	Bark	Wild	Wound healing	Dermal use of the wood ash on wounds.
2	*Acacia modesta.* Wall.	Mimosaceae	Palosa	Tree	Leaves	Wild	Tonic, stimulant	Powdered leaves or gum are taken orally at the rate of one teaspoon with milk to get instant energy. Also useful as a sex tonic.
3	*Acacia nilotica* (L.) Delile	Mimosaceae	Kekkar	Tree	Shoots	Wild	Carminative, Increase the sperm flow	Grind the newly born shoots along with some condiments (zeera) and pomegranate flowers, and use orally as carminative for infants.
4	*Allium sativum.* L.	Amaryllidaceae	Ezzha	Herb	Fruit	Cultivated	Decrease cholesterol, Bones disorders	3-5 pieces of garlic are taken orally on a daily basis to decrease the cholesterol level. Ground garlic with butter is used dermally for bone pains.
5	*Aloe barbadensis* Mill.	Asparagaceae	Zargaya	Shrub	Leaves	Wild	Wound healing	Cut the leaf and add powdered *Curcuma longa* for dermal use on wounds.
6	*Amaranthus viridis* L.	Amaranthaceae	Unknown	Herb	Leaves	Wild	Emollient	A decoction of the leaves is used dermally as an emollient and for inflammation. Root juice is also used for the same purpose.
7	*Ampelodesmos mauritanicus* (Poir.) T. Durand & Schinz	Poaceae	Khaas	Herb	Leaves	Wild	Vermicides	Leaves boiled in water and used orally.
8	*Artemisia maritima* L.	Asteraceae	Jhaan	Herb	Flowers	Wild	Vermicides	Grind the dried florets or flowers and take 1–2 tea spoons orally for intestinal worms.
9	*Arundo donax* L.	Poaceae	Kalam	Herb	Rhizome	Wild	Diuretic	Burn the underground part. The resultant ash is boiled with water, which is filtered then for oral uptake.
10	*Asparagus adscendens* Roxb.	Asparagaceae	Unknown	Herb	Rhizome	Wild	Carminative, Demulcent	Grind roots and make powder. Take this powder orally at the rate of one teaspoon.
11	*Bambusa bambos* (L.) Voss	Poaceae	Baanss	Herb	Leaves	Cultivated	Expectorant	Extract the juice from the leaves and take orally along with honey.
12	*Bauhinia variegata* L.	Papilionaceae	Kachnaal	Tree	Flowers	Cultivated	Carminative	Grind the dried flowers for oral uptake.
13	*Brassica oleracea* L.	Brassicaceae.	Gobee	Herb	Leaves	Cultivated	Vegetable, arthritis	Boil the leaves in water till it becomes greasy for dermal use on arthritis.
14	*Brassica rapa* L.	Brassicaceae	Shaljam	Herb	Whole plant	Cultivated	Blood purifier, appetizer,	Make as a pickle for an appetizer. Cook it as a vegetable, which helps in blood purification.
15	*Bryophyllum pinnatum* (Lam.) Oken	Crassulaceae	Zakham-hayat	Shrub	Whole plant	Wild/cultivated	Vermicide	Boil 10 grams of the plant in water and grind. Orally taken for 7 days on an empty stomach to kill the intestinal worms.
16	*Calotropis procera* (Aiton) Dryand	Apocynaceae	Spalmaka	Shrub	Milky extract	Wild	Dermatitis, antiseptic	Cut into portions to secrete the milky juice, which is used dermally for the curing dermatitis. Also used as an antiseptic.
17	*Cannabis sativa* L.	Cannabaceae	Bhaang	Shrub	Seeds, leaves	Wild/cultivated	Analgesic	Boil leaves and seeds in water and then spray the water dermally on painful areas.
18	*Capparis aphylla.* Roth, Nov.	Capparaceae	Karrir	Tree	Wood	Wild	Low back pain	Smolder the wood to charcoal, add 2–3 gram of this charcoal into the cooking oil to make a paste that is used dermally for back pain.
19	*Caralluma tuberculata* N.E.Br.	Apocynaceae	Pawoona	Shrub	Whole plant	Wild	Anti-diabetic, decrease cholesterol.	Cook it like a vegetable that is taken orally for diabetes treatment. Also, eat directly as a salad for sliming and for diabetic purposes.
20	*Cassia fistula* L.	Caesalpiniaceae	Garda nail	Tree	Fruits, leaves	Cultivated	Febrifuge, Purgative	Boil leaves and flowers as vegetables. Eat 2–4 fruits over 3 days for constipation.
21	*Centaurea solstitialis* L.	Asteraceae	Barham dandi	Herb	Whole plant	Wild	Febrifuge	2 spoons of powdered form are taken orally with water 3 times a day for 3 days.
22	*Chirita asperifolia* (Blume) B.L.Burtt	Gesneriaceae	Cherita	Herb	Leaves, flowers	Wild	Febrifuge	Take 25 grams of aerial part and boil it like green tea for reducing fever.
23	*Chenopodium album* L.	Amaranthaceae	Surma	Herb	Leaves, root	Wild	Laxative, Jaundice and urinary diseases	Extract the juice from their leaves, which is taken orally as a laxative.
24	*Chenopodium ambrosioides* L.	Amaranthaceae	Unknown	Herb	Fruits	Wild	Dyspepsia	The dried ripe fruits are crushed into powder form, which is taken orally with water.
25	*Cicer arietinum* L.	Papilionaceae	Chana	Herb	Fruits or grains	Cultivated	Ethno veterinary, skin itching	Grind their grains and massage this flour dermally on the itching places. Also used for ethno-veterinary purposes.
26	*Cichorium intybus* L.	Asteraceae	Bhangaara	Herb	Whole plant	Wild	Carminative	The whole plant is used for carminative purposes.
27	*Citrullus colocynthis* (L.) Schrad	Cucurbitaceae	Indrine	Herb	Whole plant	Wild	Arthritis, head ache	Cook the plant or fruit in olive oil and massage into joints or head.
28	*Convolvulus arvensis* L.	Convolvulaceae	Parvateyee	Herb	Root	Wild	Purgative	Dried roots are grinded for oral uptake of 1–2 spoons.
29	*Cordia gharaf* Ehrenb. ex Asch.	Boraginaceae	Lasora	Tree	Fruit	Wild/cultivated	Asthma, expectorant	Dried fruits are used orally for the treatment of several diseases.
30	*Coriandrum sativum* L	Apiaceae	Dhania	Herb	Leaves	Cultivated	Carminative	Roast their leaves and take with water orally.
31	*Cucumis sativus* L.	Cucurbitaceae	Kera	Herb	Fruit	Cultivated	Febrifuge, stomach	Dermal use of grinded fruit on the lower part of the foot to treat fever. Also good for digestion.
32	*Curcuma longa* L.	Zingiberaceae	Kurkaman	Shrub	Rhizome	Cultivated	Analgesic, Flu and nasal congestion	Powder form is mixed with lime and dermally used on the painful area. Put powder form on the burning coal and inhale the smoke to instantly relieve nasal congestion.
33	*Cuscuta reflexa* Roxb.	Convolvulaceae	Akas bail	Herb	Whole plant	Wild	Wound healing, analgesic	Grind the plant in an adequate amount and cook it in the oil for dermal use on wounds.
34	*Cymbopogon schoenanthus* (L.) Spreng.	Poaceae	Kana	Herb	Whole plant	Wild	Dysentery, vermicides	Boil the leaves in water and the juice is taken orally as a vermicide.
35	*Cymbopogon citratus* (DC.) Stapf	Poaceae	Lemon grass	Herb	Leaves	Wild/cultivated	Febrifuge, Flu	Boil the leaves in water for 5 minutes and add water to the milk for oral use.
36	*Cynodon dactylon* var. *coursii* (A. Camus) J.R. Harlan & de Wet	Poaceae	Owshoo	Herb	Whole plant	Wild	Smallpox, bloody piles	Grind it along with *Curcuma longa* and rice. Use the mixture for smallpox. For piles treatment, grind it with *Cannabis sativa* leaves, add milk and use orally 2 times a day.
37	*Dalbergia sissoo* DC.	Papilionaceae	Sheesham	Tree	Leaves	Wild/cultivated	Mental disorder	Take 10 g leaves, add 3 pieces of black pepper and grind for oral use.
38	*Datura stramonium* L.	Solanaceae	Dhatoora	Herb	Roots, seeds	Wild	Asthma, expectorant	Roast the leaves and inhale the smoke for asthma. Seeds are used as expectorant. Excess use can be lethal.
39	*Digera muricata* (L.) Mart.	Amaranthaceae	Unknown	Herb	Leaves, shoots	Wild	Urinary tract infection	Leaves and shoots are taken orally as a vegetable to treat urinary tract infection.
40	*Dodonaea viscosa* Jacq.	Sapindaceae	Sanatha	Shrub	Leaves	Wild/cultivated	Rheumatism, swelling and burns	Grind the leaves and add small amount of water to make fine paste for dermal use.
41	*Echinops echinatus* Roxb.	Asteraceae	Ont katara	Herb	Roots	Wild	Liver disease	Root is mixed with vinegar to make tea for oral use.
42	*Eriobotrya japonica* (Thunb.) Lindl.	Rosaceae	Alokaat	Tree	Fruits	Cultivated	Produce the fresh blood, stop the bleeding	Take simply their fruits orally for management of several diseases.
43	*Eugenia jambolana* Lam.	Myrtaceae	Jaman	Tree	Fruits, seeds	Cultivated	Antidiabetic, stomach problems	For stomach problems, grind the dried non-edible portion of fruits for oral uptake at a rate of 1–2 spoons daily for 3 days. Powder is also used for the treatment of diabetics.
44	*Euphorbia helioscopia* L.	Euphorbiaceae	Purporai	Herb	Shoot	Wild	Skin disease	Grind the dried shoots to powder for dermal use on the skin.
45	*Euphorbia hirta* L.	Euphorbiaceae	Unknown	Herb	Whole plant	Wild	Carminative, expectorant	Extract of milky juice is used orally for infants for both diseases.
46	*Euphorbia tirucalli* L.	Euphorbiaceae	Tohaar	Tree/shrub	Extract	Wild	Piles treatment	Extract their juice, add flour to it and make small tablets for oral use.
47	*Fagonia arabica* L.	Zygophyllaceae	Dhamasa	Shrub	Whole plant	Wild	Febrifuge, expectorant	Paste it with dried grapes and boil the mixture in order to make a tea for oral use.
48	*Fagonia cretica* L.	Zygophyllaceae	Spelaghzai	Herb	Whole plant	Wild	Cooling agent, scabies treatment	Grind the whole plant in water and filter it to remove the solid contents and then take 1 glass of it orally.
49	*Fagonia indica* Burm.f.	Zygophyllaceae	Spelaghzai	Herb	Whole plant	Wild	Purgative	Grind the whole plant and take 2–3 spoons orally for purgative purposes.
50	*Ficus carica* L.	Moraceae	Barrh	Tree	Leaves	Wild	Wound healing	Burn the leaves and the ash is sprayed on the wounds dermally.
51	*Ficus elastica* Roxb. ex Hornem.	Moraceae	Unknown	Tree	Leaves	Cultivated	Wound healing	Bark decoction is generally used for wound healing effect.
52	*Ficus religiosa* L.	Moraceae	Peppal	Tree	Bark, leaves	Cultivated	Stomach problems, wounds healing	Burn the bark and make powder from it. Take 5 grams of it orally with water for diarrhea; leaves are used for wound healing.
53	*Fumaria indica* (Hausskn.) Pugsley	Papaveraceae	Pith-panra	Herb	Whole plant	Wild	Blood purifier, Febrifuge	Extract their juice and take orally for purification of blood. Its tea is used for fever.
54	*Grewia asiatica* L.	Malvaceae	Falsa	Tree	Fruits	Wild/cultivated	Diabetics, cooling agent	Simply eat their fruits to help diabetics. Also provide cooling sensation.
55	*Justicia adhatoda* L.	Acanthaceae	Unknown	Shrub	Leaves	Wild	Rheumatism, stomachache	Grind the leaves and mix it with honey. The paste is used dermally around the swelling.
56	*Lactuca sativa* L.	Asteraceae	Salad	Herb	Leaves	Cultivated	Blood purifier	Simply use as a salad for blood purification.
57	*Lallemantia royleana* Benth.	Lamiaceae	Balango	Herb	Seeds	Wild	Sexual purposes, carminative	Eat seeds up to 2–5 gram to increase sperm capability. Also used as carminative.
58	*Lawsonia alba* Lam.	Lythraceae	Mehndi ka poda	Tree	Flowers	Cultivated	Sexual purpose	Cooking of the flowers along with meat is useful in increasing sexual power.
59	*Melia azedarach* L.	Meliaceae	Bankara	Tree	Seeds	Wild/cultivated	Piles treatment	Eat seed’s internal portion of 2–3 seeds only, but do not exceed as they may be lethal.
60	*Mentha piperita* L.	Lamiaceae	Podina	Herb	Leaves	Cultivated	Carminative	Make tea from their leaves and use orally 4–5 times a day.
61	*Mirabilis jalapa* L.	Nyctaginaceae	Gul-e-abassi	Herb	Root, flowers	Wild	Piles treatment, blood purifier and sexual purpose	Their roots are cooked with meat to increase sperm production and blood purification. Powdered flowers are used orally for piles treatment.
62	*Momordica charantia* L*.*	Cucurbitaceae	Karela	Shrub	Leaves	Cultivated	Vegetable, diabetics and hepatitis	As a vegetable, it’s useful for diabetics and hepatitis.
63	*Monotheca buxifolia* (Falc.) A. DC.	Sapotaceae	Gurgura	Tree	Fruits, leaves	Wild/ Cultivated	Purgative, Refrigerant	Make juice of their parts and use orally as a purgative and cooling agent.
64	*Moringa oleifera* Lam.	Moringaceae	Sohanjna	Tree	Root	Cultivated	Kidney-stone, vermicides	Cut their roots and boil in water. Add milk and drink for kidney stones and worms.
65	*Morus alba* L.	Moraceae	Shah –toot	Tree	Fruits	Cultivated	Heart, Liver tonic	Eat their fruits, which provide the energy to the heart and liver.
66	*Morus nigra* L.	Moraceae	Tooth-siah	Tree	Leaves, root	Wild/ Cultivated	Analgesic	Boil their leaves and roots in order to make tea for oral use.
67	*Musa acuminata* Colla.	Musaceae	Kela	Tree	Fruit	Cultivated	Menstruation, antidiabetic	Juice of the fruit is mixed in yogurt for oral uptake during menstruation. In antidiabetic case, roast and powder the flower for oral use.
68	*Nannorrhops ritchiana* (Griff.) Aitch.	Arecaceae	Mazara	Shrub	Leaves	Wild	Carminative, veterinary	Mostly their leaves are used to boil. The juice is then used orally.
69	*Nerium oleander* L.	Apocynaceae	Kanir	Shrub	Roots	Cultivated	Sexual purpose, strengthen the penis	Cut the root into small pieces and then boil along with milk and pour into the thin cloth and extract like butter for oral use in adequate amounts for sexual purposes.
70	*Nyctanthes arbor-tristis* L*.*	Oleaceae	Haar singhar	Shrub	Flowers	Wild/cultivated	Cough, antipyretic	Take 6 fresh leaves and grind in water with half a gram of ginger and take orally.
71	*Ocimum basilicum* L.	Lamiaceae	Takhm-rehan	Herb	Seeds	Wild	Blood purifier	Place the seeds in water to soften and enlarge, then take orally.
72	*Olea ferruginea* (Sol.) Steud.	Oleaceae	Zaiton	Shrub	Whole plant	Wild	Toothache, antidiabetic	Make powder of it and then take 1 teaspoon for 45 days orally on an empty stomach, which is helpful in uncontrolled diabetics.
73	*Opuntia triacantha* (Willd.) Sweet	Cactaceae	Zaqqoom	Herb	Leaves	Wild	Dermatitis	Extract their mucilaginous material, which is found in between leaves. Add cooking oil, make a paste and use dermally.
74	*Oxalis corniculata* L.	Oxilidaceae	Tarookay	Herb	Leaves, root	Wild	Stomach, wound healing and Anthelmintic	Extract juice from fresh leaves and use orally against stomach troubles. Leaves are used as vegetables. Crushed leaves are dermally used on wounds. Decoction of root is anthelmintic.
75	*Papaver somniferum* L.	Papaveraceae	Opium	Shrub	Fruit, leaves	Wild/ Cultivated	Analgesic, narcotics	Boil the water and add the extract of opium to it and take 1–2 spoons orally of this syrup.
76	*Peganum harmala* L.	Zygophyllaceae	Spelaanee	Herb	Seeds	Wild	Psycho-spiritual purposes	Put it on burning coal in order to produce smoke, which is used locally to repel evils.
77	*Pennisetum americanum* (L.) Leeke	Poaceae	Bajra	Herb	Grain	Cultivated	Carminative	Tie the grains in the piece of cloth, heat it and place in the abdominal region to combat pain.
78	*Periploca aphylla* Decne.	Asclepiadaceae	Baradda	Shrub	Shoots	Wild	Tumors, swellings	Generally their milky juice is extracted and then used dermally for tumors.
79	*Phoenix dactylifera* L.	Arecaceae	Khajjor	Tree	Fruits, seeds	Wild/ Cultivated	Stomach, liver tonic and carminative	Ripened fruit is useful for liver and stomach. Seeds are crushed to make a powder, which is used orally as a carminative.
80	*Plantago major* L.	Plantaginaceae	Barthang	Herb	Leaves	Wild	Dental pain	Boil leaves in water and make tea. Cool and wash the mouth.
81	*Portulaca oleracea* L*.*	Portulacaceae	Kulfa-ssag	Herb	Leaves	Cultivated	Antidiabetic	Cook like a vegetable. Do not heat it too much. The color must remain light green.
82	*Psidium guajava* L.	Myrtaceae	Amrood	Tree	Fruit	Cultivated	Purgative	Whole fruit is eaten simply as a purgative.
83	*Punica granatum* L.	Lythraceae	Anar	Tree	Fruit	Cultivated	Febrifuge, vermicide	Grind the fruit and orally take 1 spoon 2–3 times a day to kill intestinal germs. For fever, make pomegranate juice, add a little opium, then add sugar for and take orally.
84	*Raphanus sativus* L.	Brassicaceae	Mooly	Herb	Whole plant	Cultivated	Stomach problems, break the kidney stone and hepatitis	Eating simply is helpful in digestion and for the treatment of kidney stones. Boil their leaves in water and add sugar for oral uptake to treat hepatitis.
85	*Rhazya stricta* Decne.	Apocyanaceae	Ghandaryee	Shrub	Root	Wild	Analgesic	Boil roots in water for 10 minutes and cool. Wash teeth with this for pain relief.
86	*Ricinus communis* L*.*	Euphorbiaceae	Arand	Shrub	Fruits, leaves	Wild	Analgesic	Heat the leaves and fruits for the release of oil, which is spread on the desired place.
87	*Saccharum officinarum* L.	Poaceae	Gana	Shrub	Fruit	Cultivated	Strengthen the teeth’s, blood purifier and expectorant	Remove the upper portion and then cut down into small pieces and chew, which strengthens the teeth and cures others diseases.
88	*Salvadora persica* L.	Salvadoraceae	Miswak Tree	Shrub	Bark	Wild/ Cultivated	Blood purifier	Bark is commonly used as a purifying agent.
89	*Salvia aegyptiaca* L.	Lamiaceae	Balango	Herb	Small grains	Wild	Sexual purpose, male fertility	Eat directly. Small grains increase sperm count and thicken the sexual fluid. Also used for the treatment of infertile parents.
90	*Silybum marianum* (L.) Gaertn.	Asteraceae	Ont katara	Herb	Whole plant	Wild	Liver disease, carminative	Cut the roots, add vinegar and make tea, which is used orally for liver disease and for carminative purposes. Cut the roots and add vinegar in order to make pickle.
91	*Sisymbrium irio* L*.*	Brassicaceae	Kharkasai	Herb	Seeds	Wild	Febrifuge, expectorant	Seeds are used as an expectorant and used externally as a stimulating poultice. Seeds also used orally to reduce fever.
92	*Sisymbrium officinale* (L.) Scop.	Brassicaceae	Khob-kalah	Herb	Whole plant	Wild	Febrifuge, expectorant	Dry the plant into powder form, which is used orally for typhoid fever.
93	*Solanum nigrum* L.	Solanaceae	Makko	Shrub	Leaves, Root	Wild	Cancer treatment, sedative	Grind the leaves and add maize flour in equal quantity and mix both for oral treatment in the case of cancer treatment. Boil roots in water for making tea and use as a sedative.
94	*Solanum pseudocapsicum* L.	Solanaceae	Kuty lala	Herb	Leaves	Wild	Arthritis	Grind leaves and make a paste. Put on joints for the treatment of arthritis.
95	*Solanum surattense* Burm. f.	Solanaceae	Maraghareye	Herb	Fruits	Wild	Foot cracks	Cut fruits into two pieces, and massage on the foot cracks.
96	*Spinacia oleracea* L.	Amaranthaceae	Palak	Shrub	Leaves	Cultivated	Cooling agent	Boil 5–8 leaves in water and take orally for calming the stomach.
97	*Tamarix aphylla* (L.) H.Karst.	Tamaricaceae	Ghazz	Tree	Leaves	Wild	Smallpox, flatulence	Simply burn the leaves and take their decoction for the treatment of smallpox.
98	*Thuja occidentalis* L.	Cupressaceae	Sarwa	Tree	Leaves	Cultivated	Dental pain	Boil the leaves in the water and wash mouth for the relief of dental pain.
99	*Thymus vulgaris* L.	Lamiaceae	Zanglee podina	Shrub	Leaves	Wild	Flatulence	Grind the dried leaves and take 1 spoon orally with curd for calming and flatulence.
100	*Eclipta prostrata* L.	Asteraceae	Bhangaara	Herb	Leaves	Wild	Blood purifier	Eat 6–7 leaves orally for blood purification.
101	*Typha angustifolia* L.	Typhaceae	Dheela	Herb	Leaves	Wild/cultivated	Tonic	Dry the leaves and ground into flour or eaten as a cooked vegetable for tonic purposes.
102	*Vetiveria zizanioides* (L.) Nash	Poaceae	Khaas/cus cus grass	Herb	Root	Wild	Analgesic	Grind the root in water and massage the paste dermally on the head for pain relief.
103	*Vitis vinifera* L.	Vitaceae	Angoor	Shrub	Fruits	Cultivated	Carminative	Eat the fruit for carminative purpose.
104	*Withania somnifera* (L.) Dunal	Solanaceae	Shahpiangay	Shrub	Fruits, seeds	Wild	Carminative	Put 2–3 seeds or fruits into the water and then eat as a carminative.
105	*Ziziphus jujuba* Mill.	Rhamnaceae	Jangly-bera	Tree	Fruits	Wild	Intestinal, blood purifier	Eat the fruit, which is helpful in the treatment of diarrhea as well as for blood purification.
106	*Ziziphus mauritiana* var. *abyssinica* (Hochst. ex A. Rich.) Fiori	Rhamnaceae	Onaab	Tree	Fruits	Wild/cultivated	Blood purifier, smallpox and expectorant	Tea is made from the fruits, which is used individually or in combination with other drugs for curing various diseases.
107	*Ziziphus nummularia* (Burm. f.) Wight & Arn*.*	Rhamnaceae	Bair	Tree	Fruit, Root	Wild/cultivated	Blood purifier, stomach disorder and carminative	Roast the fruit and eat for the treatment of stomach problems. Take 5 grams of root and 7 pieces of black pepper, grind and take orally thrice a day for diarrhea and abdominal pain.

**Figure 2:**
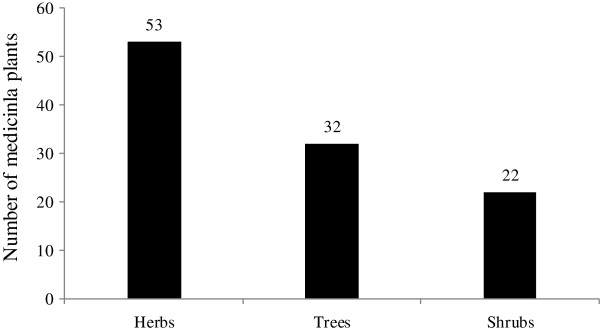
Life form distribution of ethnomedicinal plants.

Figure 2 shows that herbs (53) and trees (32) are the most common life-form of the plants described by healers as having medicinal properties. The higher use of herbs for medicinal purposes in the study area may be due to their ease of collection, higher abundance and high effectiveness in the treatment of ailments in comparison to other life-forms [9], while in other regions it may also be due to seasonal variability or differences in socio-cultural beliefs and practices of healers. With regard to trees, their extensive use in the preparation of ethnomedicines might be linked to their ability to withstand long dry seasons, thus resulting in their availability throughout the year in arid and semi-arid areas [28].

Results indicate that most of the study plants (55%) are collected from the wild to treat different diseases (Table 2). The basic reason behind using wild plants may be due to three reasons: i) The inhabitants of the region are not very well off; ii) In the current situation of war they are heavily dependent on medicinal plants; or iii) Such species are readily available at minimum cost near households as compared to cultivated plants. Giday and Tilahun [29] also found that in Ethiopia the use of wild plants is more common than the use of cultivated plants, and people also use wild medicinal plants for economic purposes. As a result, in our study area, economically valuable species such as *Caralluma tuberculata* and *Nannorrhops ritchiana*[30] which have the potential to be cultivated [20] for ecological restoration and rural livelihood are under threat due to over-collection.

### Ethnomedicinal uses

**Figure 3:**
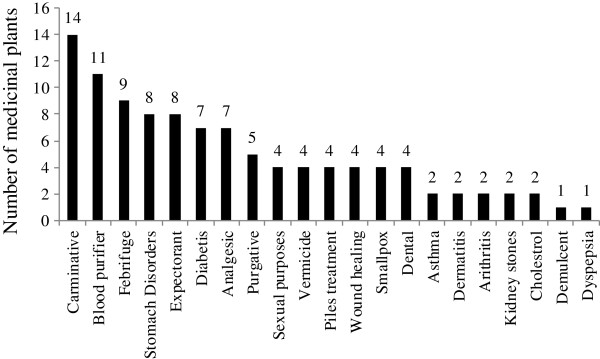
Ethnomedicinal uses in the study area.

The majority of plant species reported in the study area were used for carminative (14), blood purifying (11) or febrifuge (9) purposes, and each of the 8 species were used to treat stomach or chest problems (Figure 3). These findings are in line with other ethnobotanical studies [9,31], where most plant species were reported to be used for the treatment of chest, fever or gastro-intestinal related diseases. Such diseases may have been recently exacerbated due to the increasing armed conflict and lack of security in the area, a lack of proper sanitation, or because of wood-fuel smoke inside houses. Moreover, the majority of people in the study have little or no access to safe drinking water, which may have increased the prevalence of waterborne diseases [32]. Gastrointestinal problems are not only common in the study area but are a common issue for the whole country. According to Ribeiro *et al*. [33], such diseases can result in higher mortality rates if not treated promptly. Results of the field surveys indicate that *Acacia nilotica*, *Caralluma tuberculata*, *Convolvulus arvensis, Nannorrhops ritchiana, Withania somnifera* and some other species are used for the treatment of more than one type of ailment. Our findings are in line with the ethnobotanical studies carried out by Badshah and Hussain [34] and Khan [35], who also reported various medicinal uses for the aforementioned plants. The multiple uses for each plant serve as a strong indicator of the natural availability of a variety of therapeutic phytochemicals within the plants, and such findings may prompt further research into their medicinal application.

**Figure 4:**
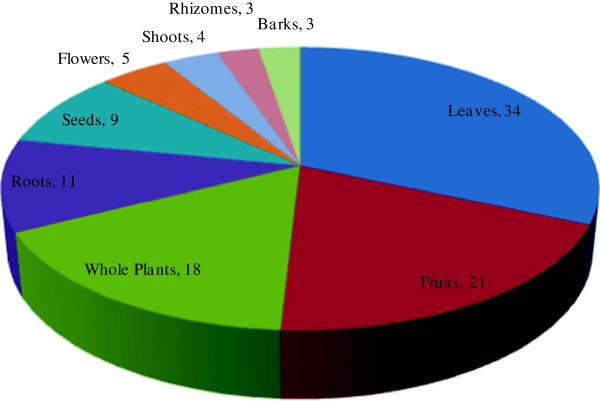
Parts of the plants (%) used in the preparation of ethnomedicines.

All parts of medicinal plants - including the leaves, stem, flower, bark, roots, fruits and seeds are used by traditional healers and local people, but the part of the plant collected for each specific purpose depends on the requirements of the user and type of plant. Figure 4 shows that 34% of plants were used for their leaves in the making of various medicinal preparations, which is easy to process into a digestible paste and have less conservational issues than the collection of roots, bark, stem or the whole plant [36]. The predominance of leaf use in the preparation of remedies has also been reported by Muthu *et al*. [37] and Kala *et al*. [38] and similar results have been reported from other areas of Pakistan [35,39,40].

Drying and pulverizing into powder, boiling for tea, juicing and pounding into a paste are the common preparation methods observed for our study area (Table 2). According to Deeba [41], grinding or crushing and boiling are the most common and effective methods of active ingredient extraction. While the majority of preparations of remedies in our study area involved the use of single plant, some of the remedies were prepared by combining different plants, for example, the powder of *Acacia nilotica* is mixed with the flowers of *Punica granatum* to be used for carminative purposes. Similarly, a mixture of leaves of *Aloe barbadensis* with the powder form of *Curcuma longa* is used to treat wounds, while the whole plant of *Cynodon dactylon* is mixed with leaves of *Cannabis sativa* and ground to powder for use against bleeding piles. According to traditional healers, complex medicines of two or more plant species are more potent than those prepared with a single species. The use of multiple therapies in traditional medicine based on combining plants has recently been shown to increase the efficacy of some herbal medicines [42]. According to Bussmann and Sharon [43], the use of more than one plant species to prepare a remedy for ailments is attributed to the additive or synergistic effects that they could have during ailment treatment. The method of drug preparation in many cases varies from individual to individual, while the same plant material for the same ailment may be prepared in different ways by different traditional healers. For example, in the present study the leaves and roots of *Oxalis corniculata* are used for stomach problems in powder form, but according to Murad *et al*. [44], the same plant is used for the same ailment in juice form in the Malakand district of Pakistan. Similarly, leaves of *Cannabis sativa* in boiled form are therapeutically very active against relieving pain in the study area, but the same plant is prepared in powder form to treat the same in the Swat region of Pakistan [5]. Such similarities in the cross-cultural usage of the traditional plant remedies are a strong indication of the bioactivity potential of the documented plant species. Table 2 shows that nearly all plant remedies are ingested orally in combination with other ingredients (vehicles) such as water, sugar, lime, wheat flour, mustard oil, honey, butter and milk to minimize the effect of the remedy’s astringent taste. It has been suggested that the use of such vehicles may dilute or reduce the relative potency of the drug [45]. However, there is no consensus on the dosage and frequency of the medication among healers because the dosage varies according to the type and severity of the illness or injury being treated.

### Traditional knowledge and age of the respondents

**Figure 5:**
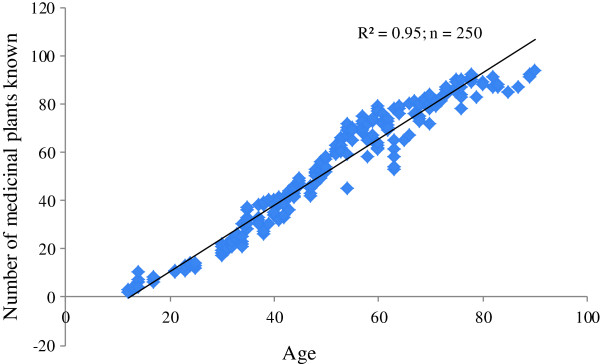
Pearson correlation between age of the respondents and the number of medicinal plants known to them.

**Figure 6:**
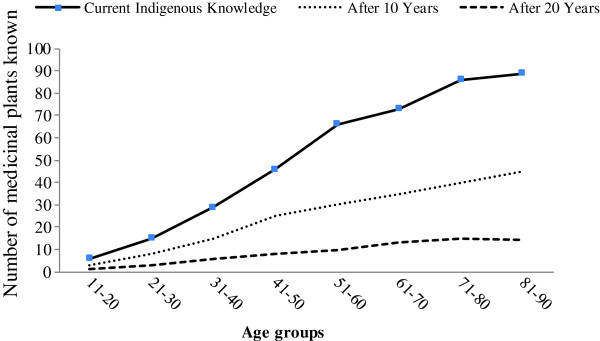
A conceptual diagram on the current and future status of indegnous knowledge.

Data analysis showed that there is a strong positive correlation (r^2^ = 0.95) between the respondent’s age and the number of medicinal plants known to them (Figure 5). In our study younger people up to 25 years old knew of approximately 15 medicinal plant species, which is far fewer than that of older people. Hussain *et al*. [46] in the South Waziristan and Parveen *et al*. [47] in the Thar Desert of India reported that people older than 30 years of age are more knowledgeable than younger ones in terms of medicinal plants and their uses. Ethnomedicinal recipes made by the local elders (collectors, traditional practitioners) are more effective than those made by young people [37,47]. This may be partly explained by recent trends of modernization that have caused the level of information being directly transmitted between generations to be greatly reduced [9], and which may lead to the eventual disappearance of such knowledge and the weakening of the relationship between people and plants. Figure 6 represents a conceptual diagram that projects a decline in indigenous medicinal plant knowledge of around 75% in the coming 20 years. Another cause of such a decline may be related to the influence of increased armed conflict in the region. A large proportion of the inhabitants of the investigated area have recently migrated to urban areas, which may exacerbate any decline in indigenous knowledge as modernization and disinterest among youth in the urban areas has rendered traditional knowledge almost extinct [12]. Traditional knowledge is now confined to the remote areas of Pakistan [14], but various remote regions are vulnerable to annual climate-change-induced water scarcity and flooding and others problems such as financial crises, high transportation costs, increased prices of consumer goods, shortages of clean water, poor social networks and terrorism, all of which encourage the migration of a large proportion of the population toward urban areas. A study conducted by Cheikhyousaf *et al*. [48] showed that most of the healers in the region gained their knowledge from their grandparents. Considering the current high levels of youth migration from rural areas, such relationships are less likely to be formed across generations, resulting in the loss of medicinal plant knowledge when traditional healers pass away. As such, the documentation of traditional knowledge on ethnomedicinal uses is regarded as a necessity to safeguard future generations and encourage further research studies.

## Conclusion

Traditional medicines serve as an integral source of rural livelihood in the study region in northwestern Pakistan, which is severely affected by armed conflict in the so-called War on Terror. The study area has plenty of medicinal plants to treat a wide spectrum of human ailments and local healers, although in decline, can be experts in the preparation of various ethnomedicinal remedies. Moreover, the use of specific plant parts, similar uses of same plants in different regions and multiple uses of single plants for the preparation of medicinal remedies suggest the prevalence of biologically active compounds across a range of medicinal plant species. Further phytochemical analysis, pharmaceutical application and clinical trials are therefore recommended in order to evaluate the authenticity of ethnomedicines to scientific standards. Indigenous knowledge on ethnomedicinal preparations persist more among older traditional healers, however, such knowledge is being lost to younger generations and continuing armed conflict in the region may further inhibit the transition of such knowledge. As such, studies on the documentation of ethnomedicines may be extended to other war-affected areas for the protection of traditional knowledge.

## Competing interests

The authors declare that they have no competing interests.

## Authors’ contributions

MA designed the research project, conducted statistical analysis and provided comments on the draft manuscript. IU conducted the field work, wrote the draft manuscript and has equally contributed as first author with MA. AT analyzed the data and helped in writing the draft of the manuscript. AU, WM, NA and ALK provided comments and suggestions on the draft manuscript. All authors have read and approved the final manuscript.
